# Thrombomodulin expression in colorectal carcinoma is protective and correlates with survival

**DOI:** 10.1038/sj.bjc.6603098

**Published:** 2006-04-18

**Authors:** A M Hanly, M Redmond, D C Winter, S Brophy, J M Deasy, D J Bouchier-Hayes, E W Kay

**Affiliations:** 1 Department of Surgery, Royal College of Surgeons in Ireland, Beaumont Hospital, Dublin, Ireland; 2 Department of Histopathology, Royal College of Surgeons in Ireland, Beaumont Hospital, Dublin, Ireland

**Keywords:** thrombomodulin, colorectal

## Abstract

Thrombomodulin (TM) is an endothelial receptor that exhibits anticoagulant, antifibrinolytic and anti-inflammatory activity by inhibiting thrombin and cellular adhesion. In this study, the expression and significance of TM was examined in primary colorectal cancer and its prognostic implications explored. TM immunostaining was performed on formalin-fixed, paraffin-embedded tissue sections, from primary lesions of 200 patients with colorectal carcinoma. Institutional Ethical approval was granted and clinical data retrieved from patients' records. All normal colonic tissue expressed TM on endothelial cells. TM tumour cell expression was demonstrated in 53 (26.5%) cases and 147 (73.5%) showed no neoplastic cell staining. On univariate and multivariate analysis TM expression on tumour cells correlated significantly with tumour stage, differentiation, Jass score and 5 year survival. TM expression decreases as overall stage and tumour size increase (*P*=0.03). In all, 91% TM positive tumours were well differentiated and 85% of TM negative tumours were poorly differentiated (*P*<0.01). Five year survival rates of patients with positive and negative TM expression were 71 and 41%, respectively. Survival rate was poorer in those patients who were TM negative compared with those who were positive (*P*<0.01). A total of 101 (50.5%) of the cases were node negative. In this group, 5 year survival rates of patients with positive and negative TM expression were 87.5 and 37.8%, respectively, demonstrating a poorer survival rate for those who are node negative and TM negative at the time of surgery (*P*<0.001). This study demonstrates that loss of TM is a key indicator in tumour biology and prognosis.

Thrombomodulin (TM) is a membrane-bound glycoprotein initially identified on vascular endothelium and later on leucocytes, mesothelium, keratinocytes and astrocytes. Expression is affected by a range of pathophysiological stimuli (Maruyama *et al*, 1985; Dittman *et al*, 1989; Ohji *et al*, 1995; Calnek and Grinnell, 1998; Ishii *et al*, 2003; Shi *et al*, 2003) and the effect of a specific stimulus varies with the cell type involved (Grey *et al*, 1998).

TM plays a role in three major processes *in vivo*: coagulation, inflammation and cell adhesion. It was discovered by its anticoagulant activity, to be an essential co-factor for thrombin-mediated activation of Protein C (Esmon and Owen, 1981, 2004) and thrombin-activated fibrinolysis inhibitor (TAFI) (de Munk *et al*, 1991), both of which demonstrate fibrinolytic activity. Through these mechanisms, the function of TM as a natural anticoagulant has been well documented.

The anti-inflammatory role of TM has been clearly demonstrated *in vivo*. It reduces restenosis (Waugh *et al*, 1999), modifies the inflammatory response (Weiler *et al*, 2001), prevents leucocyte infiltration (Ikeguchi *et al*, 2002), and these effects are mediated by a range of mechanisms (Weiler *et al*, 2001; Conway *et al*, 2002).

The role of TM in malignancy is, in contrast relatively unknown. TM expression has been immunohistochemically demonstrated in a wide variety of human tumours, (Tezuka *et al*, 1995; Hamatake *et al*, 1996; Kim *et al*, 1997; Ordonez, 1997; Tabata *et al*, 1997; Wilhelm *et al*, 1998; Zhang *et al*, 1998; Ogawa *et al*, 2000), which mostly reveal a correlation between reduced TM expression and shorter survival or increased metastases (Hamatake *et al*, 1996; Kim *et al*, 1997; Tabata *et al*, 1997; Ogawa *et al*, 2000).

The exact mechanism(s) by which TM acts within a tumour has not been clearly outlined. Anticoagulation, adhesion, differentiation and proliferation have all been suggested. What is known is that malignancy has a profound impact on the haemostatic system, with increasing recognition of the role in tumour biology of coagulation factors, anticoagulants, activators and inhibitors of fibrinolysis (Iqbal, 2000). TM may, therefore, influence tumour growth and metastasis via its role as a natural anticoagulant. TM expression is concentrated in regions of cell to cell contact (Jackson *et al*, 1995; Tezuka *et al*, 1995). It has also been demonstrated to colocalize with actin filaments (Huang *et al*, 2003) suggesting an involvement in intercellular communication or (cell–cell) adhesion. Mirroring other cell-adhesion molecules, TM expression tends to be lower in metastatic lesions than in matched primary specimens (Tezuka *et al*, 1995; Hamatake *et al*, 1996; Tabata *et al*, 1997; Ogawa *et al*, 2000).

TM is expressed during embryonic development and is associated with cell differentiation (Imada *et al*, 1990). In oral squamous epithelium, TM expression reduces in parallel with the transition from normal mucosa to dysplasia and overt carcinoma (Tabata *et al*, 1995). In tumours, therefore, decreased TM may induce loss of differentiation and enhance metastatic behaviour. *In vitro* work has demonstrated that TM reduces tumour cell proliferation and invasion (Matsushita *et al*, 1998; Zhang *et al*, 1998; Hosaka *et al*, 2000; Huang *et al*, 2003) and TM expressing cells produce smaller tumours *in vivo* (Zhang *et al*, 1998; Huang *et al*, 2003). There are, therefore, many possible mechanisms by which increased TM expression could decrease the likelihood of metastasis.

TM is not as prevalent in adenocarcinomas as in squamous cell carcinomas. In one study, <20% of adenocarcinomas of the pancreas, breast and lung were found to express TM, with no expression seen in prostatic or colonic adenocarcinoma. However, only a small number of specimens were examined (Ordonez, 1997). In a small study of 21 patients with colonic adenocarcinoma, expression on the vasculature increased as tumours progressed along the dysplasia-carcinoma sequence (Takebayashi *et al*, 1995). Expression of TM in colonic adenocarcinoma has not previously been examined in a large cohort. The aim of this study was to examine TM expression in tissue extracts of patients with primary colorectal cancer and determine if this correlates with tumour stage and prognosis.

## MATERIALS AND METHODS

**Figure 1:**
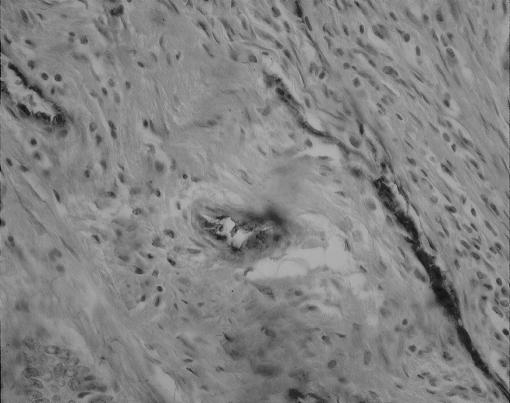
This slide demonstrates a TM negative tumour with internal positive control staining of the vessel endothelium. The slide is magnified to demonstrate this clearly.

**Figure 2:**
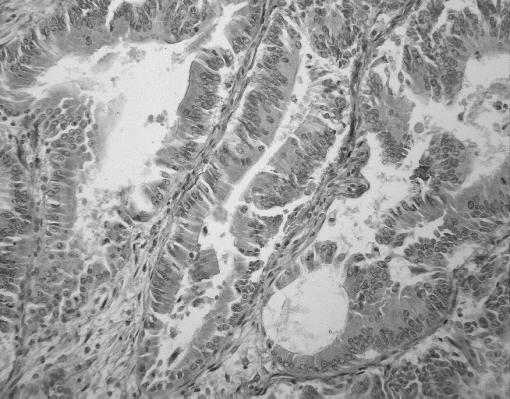
This slide demonstrates a section of colorectal tumour with +1 TM staining, internal positive control is also evident in this image.

**Figure 3:**
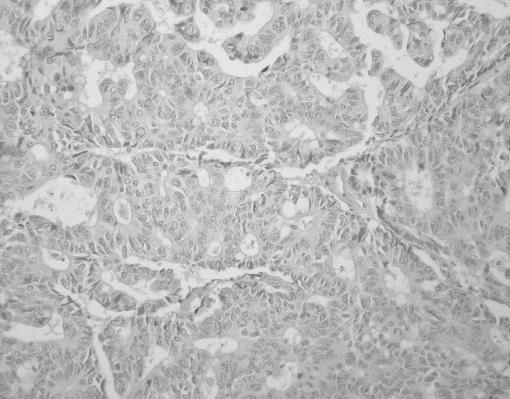
Isotype-matched primary monoclonal antibody negative control.

Specimens from 200 consecutive patients who underwent surgery for primary adenocarcinoma of the colon between 1994 and 1999 were included in this study. The median (range) age of patients was 69 (34–90) years (male 66.5%, female 33.5%). Patients with radiological evidence of metastasis, or who had preoperative chemo or radiotherapy, were excluded. Ethics approval was granted and relevant information was collected from clinical records, primary care physicians and, where necessary, patient contact. Location of tumours, overall stage and tumour stage classified by the TNM classification are outlined in Tables 1, 2 and 3, respectively. Evaluation of malignancy was based on original haematoxylin and eosin stained sections at the time of diagnosis. TM expression in the primary tumours was examined immunohistochemically. Formalin-fixed paraffin-embedded colorectal tumours were retrieved from the archives. Sections were cut at 4 *μ*m, deparaffinised and rehydrated in descending grades of alcohol (100–70%). Following heat-mediated antigen retrieval, slides were run on an automated immuno-stainer (Nexes from Ventana, AZ, USA). Primary antibody was a mouse monoclonal antibody raised against the EGF domains 4–6 of TM (Dako Glostrup, Denmark; 1 : 50 dilution). The immunostaining was developed using diaminobenzidine as a chromagen. Vascular endothelium acted as a positive internal control (Figures 1 and 2), the primary antibody was omitted for negative controls and this was confirmed using an isotype-matched primary monoclonal antibody (DAKO Cytomation M0792 at 1 : 2000 dilution) (Figure 3). Results were quantitatively evaluated by two blinded pathologists. Slides were graded according to intensity of staining on tumour cells and were classified as 0, completely negative; +1, under 10% of neoplastic cells stained; +2 10–50% of neoplastic cells stained and +3 over 50% of neoplastic cells stained. No slides demonstrated +3 staining and no statistical difference was noted between +1 and +2 staining when compared with tumour grade, stage and overall survival. Therefore, analysis was performed using two groups, TM positive and TM negative.

Statistical analysis was performed using Stats Direct (version 2.3.8. Cheshire, UK). Expression rates of TM were analysed using the Mann–Whitney *U*-test. Clinical outcome data was evaluated using the *χ*^2^ and Kaplan–Meier method. A probability of *P*<0.05 was considered statistically significant.

## RESULTS

**Figure 4:**
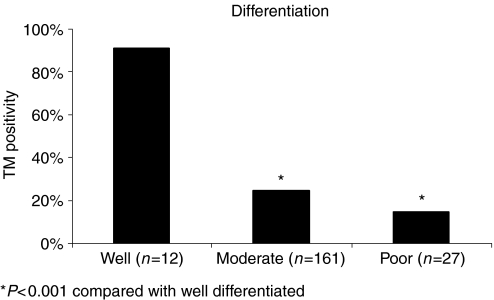
This figure demonstrates that over 90% of well-differentiated tumours were TM positive compared with 24 and 14% of moderate and poorly differentiated. Demonstrating increased TM expression in more well-differentiated tumours (Mann–Whitney *U*-test).

**Figure 5:**
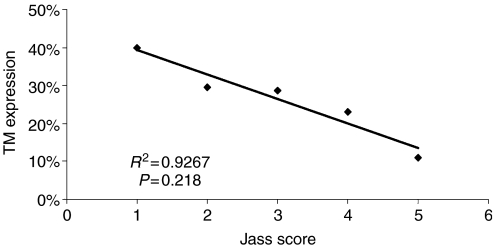
This figure demonstrates that as tumour Jass Score increases the amount of TM positive tumours exponentially decreases.

Endothelial cell TM expression was identical in normal and neoplastic colonic tissue, which acted as a positive internal control. Interestingly, however, epithelial TM expression revealed a marked difference with 7.8 and 26.5% TM positivity in normal and neoplastic colonic tissue, respectively. TM tumour cell expression was demonstrated on 53 (26.5%) tumours and 147 (73.5%) showed no neoplastic cell staining. Tables 2 and 3 compare TNM staging with positive and negative TM expression groups. This significantly demonstrates that negative TM expression correlates with advanced stage and tumour grade. On univariate and multivariate analysis tumour differentiation correlated with TM expression, with increasing expression seen in well-differentiated tumours compared with moderate and poorly differentiated (*P*<0.001) (Figure 4). There was no correlation found between site of tumour and extent of TM tumour staining. A comparison was made between TM tumour cell expression and Jass Score, expression decreasing exponentially as the Jass Score increased (Figure 5). This correlation with Jass score was not identified when comparing TM expression on the vasculature or inflammatory cells.

**Figure 6:**
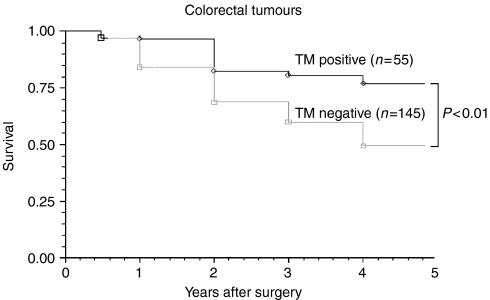
Comparison of the survival between groups which are TM positive and TM negative. The patients who were TM negative showed a significantly poorer survival than those who were TM positive.

A survival analysis was carried out using the Kaplan–Meier method. Clinical data was complete in 100% of patients. Of the 200 patients included in this study 35 (17.5%) experienced noncancer-related deaths and were considered censored in the Kaplan–Meier survival analysis. Comparisons were then made with survival and TM expression groups in the primary tumour. The 5 year survival rates of patients with positive and negative TM expression were 71 and 41%, respectively (*P*<0.01) (Figure 6).

**Figure 7:**
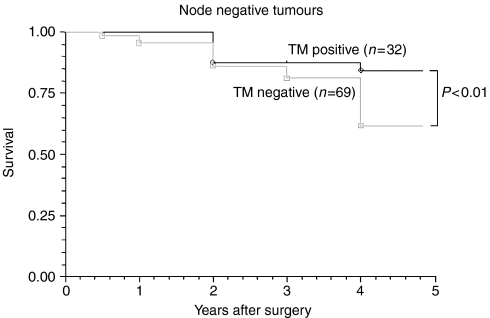
Comparison of the survival between TM positive and TM negative node negative tumours. Patients who are node negative and TM negative show a significantly poorer 5 year survival compared with those who are node negative and TM positive.

**Figure 8:**
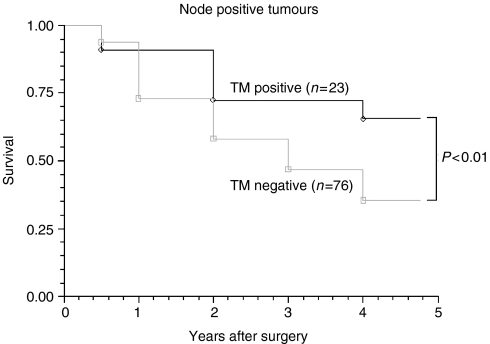
Comparison of the survival between TM positive and TM negative node positive tumours. Patients who are node positive and TM negative show a significantly poorer 5 year survival compared with those who are node positive and TM positive.

We analysed TM expression in the 101 (50.5%) patients who were node negative. The 5 year survival rates of patients with positive and negative TM expression were 87.5 and 37.8%, respectively (*P*<0.01) (Figure 7). Comparatively, we analysed those 99 (49.1%) patients, who were node positive, with 23.3 and 76.7% TM positive and TM negative, respectively. Similar to those who were node negative, 5 year survival was significantly better in those who were TM positive (Figure 8).

**Figure 9:**
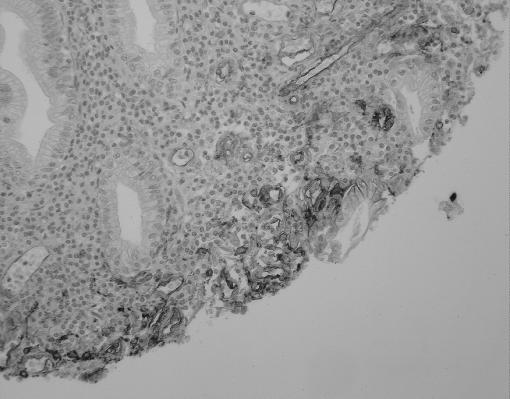
Ulcerated colorectal lesion demonstrating membranous TM staining on the inflammatory cells of the ulcer.

All ulcerated tumours expressed membranous TM on inflammatory cells within the ulcer, representing 164 (82%) of all tumours (Figure 9). TM tumour expression was similar in ulcerated and nonulcerated tumours, with 28 *vs* 26% TM positivity in ulcerated and nonulcerated tumours, respectively. Tumour staining was evenly distributed throughout ulcerated tumours and no localization of tumour cell TM expression was noted within or surrounding ulcerated areas. On further analysis patients with ulcerated tumours had poorer 5 year survival rates compared with those without ulceration, 47.1 and 61.1%, respectively (*P*<0.01). Tumours within the ulcerated group, which expressed TM, demonstrated a similarly improved survival to those in the nonulcerated group. Poorer overall survival in ulcerated tumours could not then be correlated with TM expression.

## DISCUSSION

TM is known to be produced predominately by vascular endothelial cells (Esmon, 1989; Lindahl *et al*, 1993), effecting local as well as systemic action, suggested by its presence in the plasma of healthy individuals (Lindahl *et al*, 1993). It has been widely investigated as a marker of inflammatory and vascular disease with high plasma and endothelial levels mirroring severity (Weiler and Isermann, 2003). More recently, TM expression was demonstrated constitutively by some epithelial carcinomas, with decreased expression associated with metastases and high recurrence rates (Iino *et al*, 2004). It was the aim of this study to investigate the expression of TM in a large cohort of primary colorectal adenocarcinomas and to correlate this expression with clinical data.

Tumour cell staining was inversely and significantly correlated with overall stage (Table 2), T stage (Table 3), differentiation (Figure 4), Jass Score (Figure 5) and 5 year survival (Figure 6), suggesting that the presence of TM on colorectal tumour cells inhibits differentiation and may regulate tumour growth and metastasis. TM expression was associated with improved 5 year survival in both node negative and node positive patients. Tumours without TM expression were more likely to be poorly differentiated and have a worse prognosis (*P*<0.01).

Ulcerated tumours had similar neoplastic cell TM expression compared with nonulcerated tumours. However, ulcerated tumours demonstrated greater TM expression predominantly on inflammatory cells of the ulcer. Ulcerated tumours were associated with poorer survival compared with nonulcerated tumours (*P*<0.01), despite no significant difference in TM expression, tumour grade or TNM stage. Ulcerated tumours are known to have a worse prognosis compared with nonulcerated tumours, but TM positivity within the ulcerated group was associated with a better prognosis. Poorer survival rates of ulcerated tumours are therefore thought to be independent of TM tumour cell expression.

TM expression on colorectal tumour cells correlated with overall improved survival in this study. In addition, node negative (Dukes' B) and node positive (Dukes' C) tumours independently demonstrated significantly improved survival rates with TM positive *vs* TM negative expression. It would seem, therefore, that similar to squamous and other epithelial carcinomas, neoplastic TM expression is protective in primary colorectal adenocarcinomas. However, unlike squamous cells where TM is expressed under normal conditions (Mizutani *et al*, 1996; Yonezawa *et al*, 1987) only 7.8% of normal colonic epithelial cells expressed TM. Therefore, while colonic epithelial cells do not produce TM under normal conditions, they must be phenotypically capable of doing so in certain environments.

Future work on the manipulation of TM and understanding further its role in proliferation, differentiation and adhesion, will be important in clarifying the function of TM in tumour growth and metastases. There are already many known inducers of TM expression (Hanly *et al*, 2005) and by understanding the role of TM in adenocarcinomas, these inducers may provide future potential therapies to modulate tumour behaviour and decrease metastases.

## Figures and Tables

**Table 1:** Distribution of primary tumours within the colon

**Site of tumour**	**Number of tumours**
Recto-sigmoid	82
Left colon	46
Transverse colon	8
Right colon	31
Caecum	33

**Table 2:** Comparison of overall colonic tumour stage as defined by the American joint committee on colorectal cancer (AJCC), with positive and negative TM expression groups

**Stage**	**No. patients**	**TM-positive (%) (*n*=56)**	**TM-negative (%) (*n*=144)**	***P*-Value**
I	17	9 (52.9%)	8 (47.1%)	^*^P=0.08
II	84	23 (27.4%)	61 (72.6%)	
III	99	24 (24.2%)	75 (75.8%)	
				

^*^Overall analysis of variance. ^*^Comparison of stage I vs II → P=0.06, I vs III → P=0.025, II vs III → P=0.63.

**Table 3:** Correlation of tumour stage (AJCC) with TM positive and negative groups

**Tumour grade**	**No. of patients**	**TM-positive (%) (*n*=56)**	**TM-negative (%) (*n*=144)**	***P*-Value**
T1	2	1 (50%)	1 (50%)	^*^P=0.03
T2	28	14 (50%)	14 (50%)	
T3	147	35 (23.8%)	112 (76.2%)	
T4	23	6 (26.1%)	17 (73.9%)	
				

^*^Overall analysis of variance.
